# Leptin Promotes White Adipocyte Browning by Inhibiting the Hh Signaling Pathway

**DOI:** 10.3390/cells8040372

**Published:** 2019-04-24

**Authors:** Jie Wang, Jing Ge, Haigang Cao, Xiaoyu Zhang, Yuan Guo, Xiao Li, Bo Xia, Gongshe Yang, Xin’e Shi

**Affiliations:** Key Laboratory of Animal Genetics, Breeding and Reproduction of Shaanxi Province, Laboratory of Animal Fat Deposition and Muscle Development, College of Animal Science and Technology, Northwest A&F University, Yangling 712100, Shaanxi, China; wangjie93@nwafu.edu.cn (J.W.); jingjing1991@nwafu.edu.cn (J.G.); caohaigang@nwafu.edu.cn (H.C.); xiaoyuzhang08@163.com (X.Z.); Guoyuanworkgz@163.com (Y.G.); nice.lixiao@gmail.com (X.L.); imed23@nwafu.edu.cn (B.X.); gsyang@nwafu.edu.cn (G.Y.)

**Keywords:** leptin, Hh signaling pathway, adipocyte, browning

## Abstract

Leptin is an important secretory protein that regulates the body’s intake and energy consumption, and the functions of the Hh signaling pathway related to white adipocyte browning are controversial. It has been reported that leptin plays a critical role in adipogenesis by regulating the Hh signaling pathway, but whether there is a functional relationship between leptin, the Hh signaling pathway, and adipocyte browning is not clear. In this research, mouse white pre-adipocytes were isolated to explore the influence of the Hh signal pathway and leptin during the process described above. This showed that leptin decreased high fat diet-induced obese mice body weight and inhibited the Hh signaling pathway, which suggested that leptin and the Hh signaling pathway have an important role in obesity. After activation of the Hh signaling pathway, significantly decreased browning fat-relative gene expression levels were recorded, whereas inhibition of the Hh signaling pathway significantly up-regulated the expression of these genes. Similarly, leptin also up-regulated the expression of these genes, and increased mitochondrial DNA content, but decreased the expression of Gli, the key transcription factors of the Hh signaling pathway. In short, the results show that leptin promotes white adipocyte browning through inhibiting the Hh signaling pathway. Overall, these results demonstrate that leptin serves as a potential intervention to decrease obesity by inhibiting the Hh signaling pathway.

## 1. Introduction

Adipose tissue plays an important role in maintaining the body’s energy balance. Adipose tissue is an important endocrine organ; it participates in the activities of the body for physiological regulation by secreting a variety of adipose cytokines [1]. Adipocyte differentiation is regulated by multiple cell signaling pathways. In recent years, studies have demonstrated that the Hh signaling pathway plays an important role in muscle [2,3] and adipose tissue [4,5], but how it regulates white adipose tissue browning is still unknown. In addition, Choi pointed out that the combination of leptin and Lep-R can promote the activation of the Hh signaling pathway, which promotes the trans-differentiation of hepatic stellate cells (HSCs) into hepatic fibrotic cell-type HSCs (MF-HSCs) [6,7]. Later, Liu also showed that there are interactions between leptin and the Hh signaling pathway during the differentiation and maturation of adipocytes, and leptin upregulates the expression of Glis in the Hh pathway [8]. Thus, leptin may regulate adipocyte synthesis through the Hh signaling pathway.

Hh protein, a “segment polarity” gene, was first discovered in *Drosophila* by Nusslein-Volhard and Wieschaus [9]. Hh mutations can lead to *Drosophila* embryos becoming spiny, small, and globular, like a hedgehog, and thus this mutation is called “Hedgehog” [10]. The Hh signaling pathway is highly conserved among species [11], and the mechanism is complicated in mammals, which rely on primary cilia. Hh signaling is mainly regulated by the cell membrane receptors Patched (Ptc) and Smoothened (Smo). The nuclear transcription factor (glioma-related gene, Gli) family includes many multifunctional transcription factors. The Hh signaling pathway is a classic control signal transduction pathway in embryonic development, stem cell biology, and tissue homeostasis [12]. Research has shown that the abnormal activation of the Hh signaling pathway can result in tumor occurrence [13,14], with many other signaling pathways (such as Wnt, RAS, and TGF-β/BMP) co-regulating the occurrence and growth of cancer [15,16,17]. Furthermore, Hh deactivation is associated with many developmental defects and congenital malformations [18]. Therefore, the Hh signaling pathway is a therapeutic target for many types of cancers.

Leptin is a secretory protein of adipose tissue that is widely found in mammals, amphibians, reptiles, and fish. Its genetic sequence is highly conserved. It enters the brain through the blood–brain barrier, and inhibits the expression of POMC and NPY/AgRP in the hypothalamic arcuate nucleus (ARC), which suppresses appetite in the brain [19,20,21] and increases energy release. This inhibits adipocyte synthesis, and thereby reduces body weight. Therefore, leptin plays a key role in regulating the physiological processes of food intake, glucose homeostasis, and energy intake and consumption [7,22,23].

To explore the effect of the Hh signaling pathway in the process of adipocyte browning, and the relationship between the leptin/Hh signaling pathway and adipocyte browning, two compounds were used to inhibit or activate the Hh pathway, respectively. Cyclopamine (Cy), a plant-derived teratogen [24], inhibits the Hh pathway by directly binding to Smo to affect its conformation [25]. Similarly, purmorphamine (Pu) is regarded as an activator of the Hh signaling pathway by directly targeting the Smo protein [26,27]. In addition, we also used leptin recombinant proteins to treat cells. Through this study, it is possible to gain a meaningful understanding of the mechanism of the Hh signaling pathway in white adipocyte browning, and the relationship between leptin and the Hh signaling pathway, and adipocyte browning.

## 2. Materials and Methods

### 2.1. Experimental Animals

Three-week-old C57BL/6 male mice were purchased from the Medical Laboratory Animal Center of Xi’an Jiaotong University (Xi’an, China). The animal experiments were developed with reference to the Guide for the Care and Use of Laboratory Animals of China.

### 2.2. Leptin Recombinant Treatment In Vivo

To determine the role of leptin in vivo, leptin recombinant protein was injected into high fat diet (HFD) induced obesity mice. Briefly, the 3-week-old male C57BL/6J mice, after 8 weeks of an HFD (containing 60% fat, TrophicDiet, Nantong, China), were injected with leptin recombinant protein (R&D system) or phosphate buffered saline (2.5 mg/kg) into the intraperitoneal. The injections were performed for seven days, twice a day. The mice were housed in a 12:12 h light/dark cycle with free access to water and food.

### 2.3. HE Stain

Immediately after sampling, the adipose tissue was fixed with 4% paraformaldehyde. After this, the samples were dehydrated and embedded in paraffin. Sections were cut using a Nikon TE2000 microscope (Nikon, Tokyo, Japan) and standard HE staining was performed.

### 2.4. Serum Leptin Concentration Analysis

The serum leptin concentration was measured by means of a commercial canine leptin sandwich ELISA kit (Hengyuan, Shanghai, China), according to the manufacturer’s instructions. The absorbance of each well at 450 nm was measured with a microplate reader (PerkinElmer, Singapore).

### 2.5. Cell Culture and Treatment

The mice were sacrificed, and inguinal white adipose tissue (iWAT) was isolated and washed three times with PBS. The fat pad was cut into approximately 4 mm^3^ pieces. Type I collagenase was digested for 40–50 min with oscillation of water at 37 °C. The same volume of Dulbecco’s modified Eagle’s medium/Nutrient Mixture F12 (DMEM/F12) medium with 10% fetal bovine serum (FBS, Gibco) was added to terminate digestion, then the solution was sieved through 70 and 200 mesh screens after being collected in a 10 mL tube, undergoing 1800 rpm centrifugation for 7 min, abandoning the supernatant, adding the DMEM/F12 serum-free medium rinse again, 1500 rpm centrifugation for 5 min, and culturing in DMEM/F12 serum medium at 37 °C in a humidified atmosphere with 5% CO_2_. After 24 h, it was washed twice using PBS and the DMEM/F12 serum medium was replaced. After that, the culture medium was changed every two days. Confluent pre-adipocytes were subjected to brown adipocyte differentiation medium I (DMEM/F12 with 10% FBS and added 0.5 mmol/L IBMX, 1 µmol/L Dex, 5 µg/mL insulin, 1 nmol/L T3, 125 nmol/L Indol, and 1 µmol/L rosiglitazone). The cells were switched to brown adipocyte differentiation medium II (DMEM/F12 with 10% FBS and 5 µg/mL insulin, and 1 nmol/L T3) after two days. Then, the culture medium was changed every other day and collected at different time points. A 10 µmol/L concentration of activators or inhibitors, or a 100 nmol/L concentration of leptin recombinant, was added each time the brown adipocyte differentiation medium was changed. However, the activator and leptin treatment was different compared with the activator or leptin alone. In this step, a 10 µmol/L concentration of activators and 100 nmol/L concentration of leptin were added into differentiation medium II. All the liquids were combined with 100 IU/mL penicillin–streptomycin.

### 2.6. Oil Red O Staining

Oil red O was filtered and diluted (3:2) with double distilled water. The cells were washed with PBS three times after dyeing, and fixed in 4% paraformaldehyde for 30 min. After three washes with PBS, oil red O staining was undertaken for 30 min. PBS was used again for washing three times, and finally the picture was taken.

### 2.7. Bodipy and DAPI Staining

Bodipy was diluted with PBS (1:1000) and DAPI was also diluted with PBS (1:1000). The cells were washed with PBS three times after dyeing and fixed in 4% paraformaldehyde for 30 min. After washing with PBS three times, bodipy staining was undertaken for 30 min. Then, it was placed on a horizontal shaker and washed with PBS once every 5 min. After washing three times, DAPI staining was undertaken for 10 min, washing was completed another three times, and finally a picture was taken with a fluorescence microscope (Nikon, Tokyo, Japan).

### 2.8. RNA Extraction and RT-qPCR

Total RNA was extracted using the Trizol (TakaRa, Otsu, Japan) method, following the manufacturer’s instructions. mRNA was reverse transcribed with transcription kits (TakaRa, Otsu, Japan) to synthesize cDNA, and the cDNA was performed using SYBR green kits on a Step one plusTM system (Thermo Fisher, Waltham, MA, USA). The primer sequences for the genes are shown in Table 1, and the data were processed using the 2^−ΔΔCT^ method.

### 2.9. Mitochondrial DNA Content Measurement

To determine mitochondrial DNA (mtDNA) content, genomic DNA was extracted using a DNA isolation kit (Solarbio, Beijing, China). The mtDNA copy number was calculated from the ratio of the 16s rRNA and a mtDNA to the hexokinase 2 gene. The RT-qPCR primer sequences are shown in Table 1.

### 2.10. Western Blotting Analysis

The cell total protein was isolated using RIPA (Applygen Technologies Inc., Beijing, China), and protease inhibitor (CWBIO, Shanghai, China) was added into the RIPA at a ratio of 1:100. After adding RIPA to the cell culture plate, we collected the cells, then followed this by centrifuging (12,000 rpm) at 4 °C for 10 min. Protein concentrations were measured using the Thermo Scientific Pierce BCA protein assay kit (Thermo Fisher, USA), and 1/4 volume of 5× loading buffer was added to the supernatant. A total of 20 µL of protein was blotted using 10% SDS-polyacrylamide gel, and transferred to a polyethylene difluoride (PVDF) membrane (CST, Boston, MA, USA). After blocking with 5% defatted milk for 2 h, membranes were incubated with antibodies (1:1000) against Gli1 (BOSTER, Wuhan, China), Gli2 (Wanleibio, Shenyang, China), Gli3 (Wanleibio, China), PGC1α (Abcam, Cambridge, MA, USA), PPARγ (Abcam, USA), UCP1 (Abcam, USA), aP2 (Abcam, USA), Cox7a (Abcam, USA), and β-tubulin (Abcam, USA) overnight at 4 °C. The membrane HRP goat anti-mouse IgG, goat anti-rabbit IgG, or rabbit anti-goat IgG secondary antibodies (BOSTER, China) were diluted 1:3000 and incubated for 1 h. Detection was performed using chemiluminescence western blotting substrate (Santa Cruz, CA, USA), with lmage Lab analysis software (Image Lab™, Bio-Rad, Berkeley, CA, USA).

### 2.11. Statistical Analyses

All data were expressed as means ± SD, and statistical analysis was performed with GraphPad Prism 7.0. Data were analyzed using a Student’s test or one way ANOVA. A *p* value < 0.05 was considered statistically significant, and a *p* value < 0.01 was considered extremely statistically significant. (* *p* < 0.05; ** *p* < 0.01).

## 3. Results

### 3.1. Leptin May Induce White Adipose Browning through the Hh Signaling Pathway

As a fat-secreting factor, more and more studies have shown that leptin can regulate the energy metabolism of the body in several different ways [28]. Some studies have shown that leptin can also regulate browning of white adipose and adipose differentiation through the Hh signaling pathway [8,29,30]. Previous research has shown that the Hh signaling pathway can regulate the deposition of white adipose and brown adipose. However, whether leptin regulates the conversion of white fat to brown fat through the Hh signaling pathway is unclear. There are many ways to induce browning, including chronic cold exposure (4 °C for 7 d), β-adrenergic agonist treatment (CL316243, 1 mg/kg for 7 d), and a swimming exercise protocol [31]. Thus, we used the GEO-DataSets (GSE86338) to explore the correlativity between leptin and the Hh signaling pathway in white adipocyte browning.

**Figure 1:**
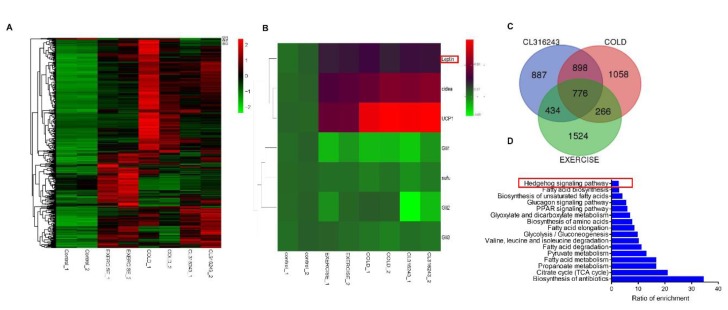
Leptin may induce white adipose browning through the Hh signaling pathway. (**A**) Heat map summarizing expression patterns of mRNA and the Hh signaling pathway regulated by browning induction. (**B**) Heat map of mRNA expression patterns of browning adipose, leptin, and the Hh signaling pathway regulated by browning induction. (**C**) Venn diagram analysis of overlap between the significant differential expression of mRNA (false discovery rate (FDR) ≤ 0.05 and absolute log2FC ≥ 1). (**D**) KEGG pathway enrichment analyses were applied based on any two groups.

After browning induction, there were 1062, 837, and 673 up-regulated and 1201, 681, and 862 down-regulated genes in the cold, CL316243, and swimming exercise treatments, respectively. These genes included Gli1, Gli2, Gli3, Sufu, and more, which all belong to the Hh pathway signaling transduction molecule. Interestingly, leptin expression was significantly increased (Figure 1A,B). In addition, we found 776 commonly regulated genes in all three conditions (Figure 1C, false discovery rate (FDR) ≤ 0.05 and absolute log2FC ≥ 1). As expected, in these mRNA we detected UCP1, Elvol3, *Cidea*, and many mitochondria-related genes. We next studied the common transcriptomic changes in any two groups and generated enriched KEGG pathways clustered by target genes of the Hh signaling pathway, for exploring its potential roles as a biomarker of browning (Figure 1D). Therefore, it is presumed that the Hh signaling pathway was dramatically induced and plays an important role in browning, and this role may be induced through leptin regulation.

### 3.2. Leptin Decreases Food Intake and Adipose Weight of High Fat Diet (HFD)-Induced Obese Mice by Inhibiting the Hh Signaling Pathway

**Figure 2:**
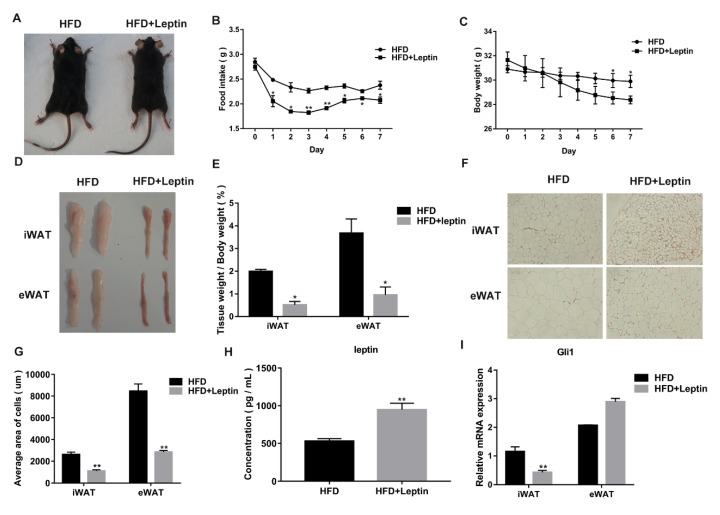
Leptin decreases food intake and adipose weight of high fat diet (HFD)-induced obese mice by inhibiting the Hh signaling pathway. (**A**) Representative image of HFD-fed mice injected with leptin recombinant protein or the control for seven days. (**B**,**C**) Daily food intake and body weight of the mice in the control and leptin recombinant protein injection groups (n = 4–6 per group). (**D**) Representative images of inguinal and epididymal white adipose tissue (iWAT and eWAT, respectively) injected with leptin recombinant protein or the control for seven days. (**E**) White adipose tissue masses were determined relative to body weight from HFD-fed mice injected with leptin recombinant protein or the control (n = 5). (**F**) Representative images of white adipose (iWAT and eWAT) stained with HE from HFD-fed mice injected with leptin recombinant protein or the control (n = 3). (**G**) Every adipose cell area in the eWAT and iWAT sections stained with HE in HFD-fed mice injected with leptin recombinant protein or the control (n = 3). (**H**) Serum leptin concentration was detected by ELIAS kit after injecting with leptin recombinant protein or the control (n = 5). (**I**) RT-qPCR was used to detect the expression level of the Hh signaling pathway marker gene *Gli1* in eWAT and iWAT after injecting with leptin recombinant protein or the control. Scale bar = 20 μm. *, *p* < 0.05; **, *p* < 0.01.

Previous research has shown that leptin can improve metabolism in ob/ob mice prone to obesity [32]. In order to study the role of leptin in obese mice, leptin recombinant protein or phosphate buffered saline was given to the HFD-induced obese mice through intraperitoneal injections. After seven consecutive days of acute injections, obese mice given the leptin recombinant protein had a lower food intake (Figure 2B) and body weight (*p <* 0.05) (Figure 2A,C), and the iWAT and eWAT weights were significantly decreased *(p <* 0.05) (Figure 2D,E). Hematoxylin and eosin (HE) stain results confirmed that, after leptin injections, iWAT and eWAT cell areas significantly decreased (*p* < 0.05) (Figure 2F,G). Serum leptin concentration was measured by ELIAS kit, and after leptin injections, serum leptin concentration was significantly increased *(p <* 0.05) (Figure 2H). RT-qPCR was used to analyze the *Gli1* expression of white adipose. Leptin recombinant protein significantly decreased *Gli1* expression in iWAT (*p* < 0.05) (Figure 2I). However, the eWAT *Gli1* expression levels were increased (*p* > 0.05) (Figure 2I). This preliminarily indicates that leptin decreases weight gain in HFD-induced obese mice through inhibiting the Hh signaling pathway, but the specific effect needs further study.

### 3.3. Hh Signaling Pathway Inhibition Promoted White Adipocyte Browning

**Figure 3:**
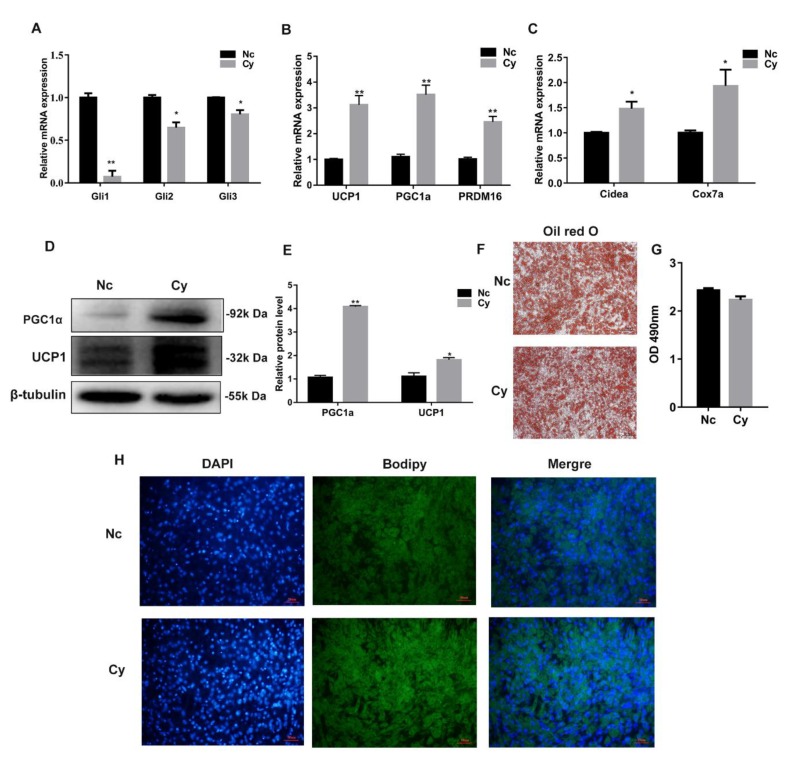
Hh signaling pathway inhibition promoted white adipocyte browning. (**A**) RT-qPCR analysis of key transcription factor gene expression of the Hh signaling pathway. (**B**) RT-qPCR was used to detect white adipose thermogenesis relative gene expression. (**C**) RT-qPCR analysis of mitochondrial marker gene expression. (**D**) Western blotting analysis of protein change of genes associated with white adipose thermogenesis relative genes. (**E**) The quantified results of the protein level. (**F**) Oil red O staining. (**G**) The result of oil red O extraction. (**H**) Bodipy staining. Data were representative of means ± SD of three independent experiments. Scale bar = 10 μm, * *p* < 0.05; ** *p* < 0.01. (Note: Nc is the cyclopamine (Cy) control, Cy was dissolved in ethanol).

Our data show that the Hh signaling pathway has an important relationship with obesity; inhibiting the Hh signaling pathway decreased the body weight of HFD mice. Therefore, we speculated that the Hh signaling pathway may affect adipose cell thermogenesis. Thus, the white adipocytes were isolated from mice inguinal tissues with browning induction cell differentiation. Cy, the Hh signaling pathway inhibitor, was used during the process. After Cy treatment, the RT-qPCR data showed that the expression of *Gli1/2/3*, the key transcription factors of the Hh signaling pathway, were significantly decreased (*p* < 0.05 or *p* < 0.01) (Figure 3A). However, the expression levels of the thermogenesis relative genes *UCP1*, *PGC1α*, *PRDM16*, and the mitochondrial genes *Cidea* and *Cox7a*, were significantly increased (*p* < 0.05 or *p* < 0.01) (Figure 3B,C). Western blotting analysis confirmed that Cy significantly increased UCP1 and PGC1α expression (*p* < 0.05 or *p* < 0.01) (Figure 3D,E). Bodipy and oil red O staining results showed that Cy reduced cell lipid droplet size after the cells were treated for 8 d (Figure 3F–H). These results indicated that inhibition of the Hh signaling pathway could promote white adipocyte thermogenesis, enhancing white adipocyte browning.

### 3.4. Hh Signaling Pathway Activation Inhibited White Adipocyte Browning

**Figure 4:**
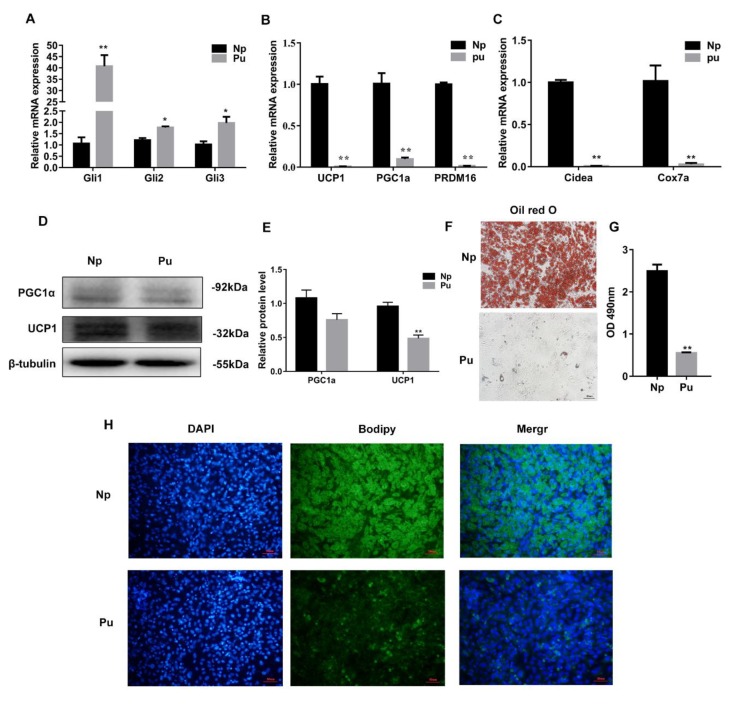
Hh signaling pathway activation inhibited white adipocyte browning. (**A**–**C**) RT-qPCR analysis of the gene expression of the Hh signaling pathway key transcription factor Glis, white adipose thermogenesis related gene expression, and expression of mitochondrial related genes. (**D**) Western blotting to detect the genes associated with white adipose thermogenesis expression. (**E**) The resulting quantity protein level. (**F**) Oil red O staining. (**G**) The result of oil red O extraction. (**H**) Bodipy staining. Scale bar = 10 μm *, *p* < 0.05; **, *p* < 0.01. (Note: Np is the purmorphamine (Pu) control, Pu was dissolved in DMSO).

To further explore the function of Hh signaling on white adipocyte browning, Pu was used to activate the Hh signaling pathway. RT-qPCR data showed that the expression levels of the Hh signaling pathway key transcription factor genes *Gli1/2/3* were significantly increased (*p* < 0.05 or *p* < 0.01) (Figure 4A). The expression of the thermogenesis related genes *UCP1*, *PGC1α*, and *PRDM16* and the expression of the mitochondrial genes *Cidea* and *Cox7a* were significantly decreased (*p* < 0.05 or *p* < 0.01) (Figure 4B,C) after white adipose cells were treated with Pu. Western blotting analysis showed that Pu significantly decreased UCP1 and PGC1α expression (*p* < 0.05 or *p* < 0.01) (Figure 4D,E). The results of bodipy and oil red O staining showed that Pu reduced lipid droplet size after the cells were treated for 8 d (Figure 4F–H). The study indicated that Hh signaling pathway activation negatively regulates white adipocyte browning.

### 3.5. The Expression Level of Gli1 Negatively Correlated with the Concentration of Leptin

**Figure 5:**
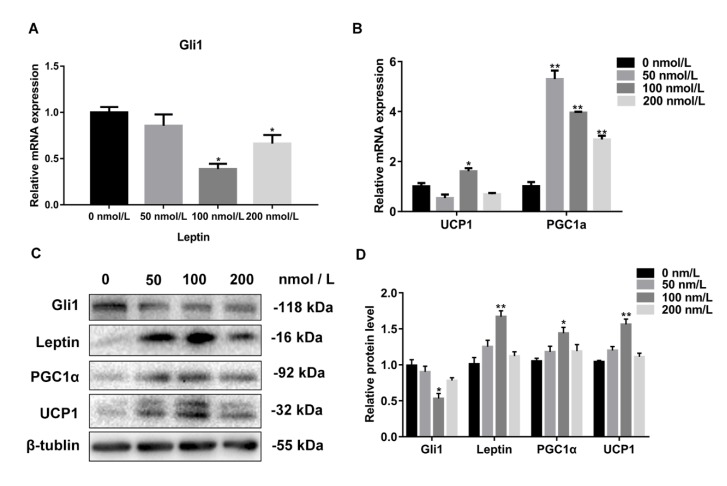
The expression level of Gli1 is negatively correlated with the concentration of leptin. (**A**) RT-qPCR was used to detect the key transcription factor Gli1 of the Hh signaling pathway. (**B**) RT-qPCR was used to detect the white adipose thermogenic related genes. (**C**) Western blotting was used to detect the expression of genes associated with the Hh signaling pathway, and thermogenic relative genes. (**D**) The resulting quantity protein level. Data are expressed as the means ± SD of three independent experiments. *, *p* < 0.05; **, *p* < 0.01.

To study the relationship between leptin and the Hh signaling pathway in white adipocyte browning, white adipocytes were isolated from mice inguinal tissue with browning induction cell differentiation. In the process of cell differentiation, the cells were treated with different concentrations of leptin. RT-qPCR was used to analyze the expression level of *Gli1*, the key transcription factor of the Hh signaling pathway. We observed that the mRNA expression of *Gli1* reduced in a concentration-dependent manner, with the increase of leptin concentration. However, the expression of *Gli1* was increased in cells treated with 200 nmol/L of leptin treatment (*p* < 0.05) (Figure 5A), and the protein expression level of Gli1 was consistent with the mRNA level (*p* < 0.05) (Figure 5C). Interestingly, the expression level of *UCP1* and *PGC1α*, the thermogenic related genes, had an opposite expression trend to that of *Gli1* in mRNA and in terms of protein levels (*p* < 0.05 or *p* < 0.01) (Figure 5B–D). This study further showed that leptin can regulate the Hh signaling pathway, and white adipocyte browning.

### 3.6. Leptin Promotes White Adipocyte Browning by Inhibiting the Hh Signaling Pathway

**Figure 6:**
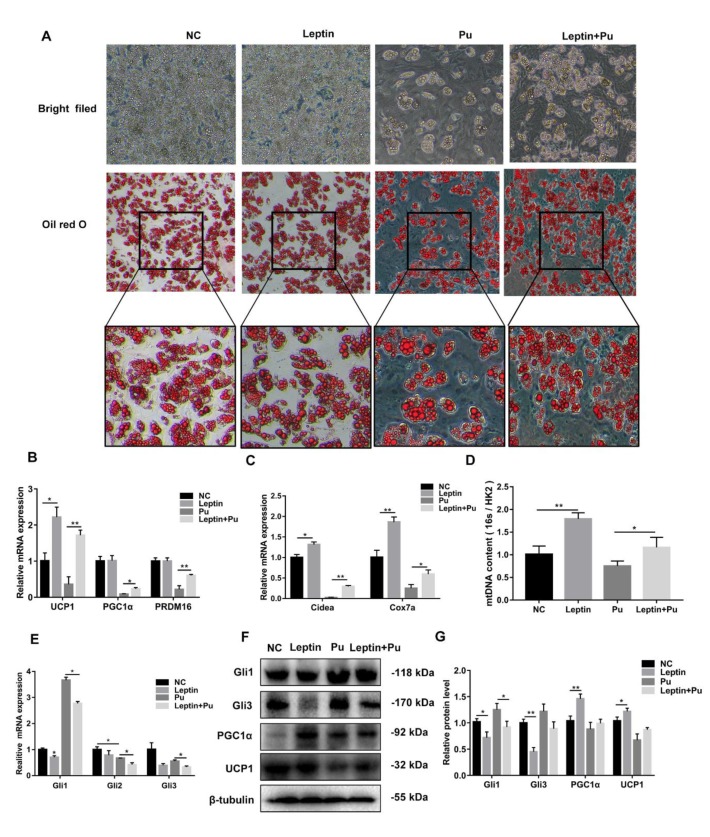
Leptin promotes white adipocyte browning by inhibiting the Hh signaling pathway. (**A**) Oil red O staining. (**B**–**E**) RT-qPCR analysis of gene expression related to thermogenic genes, mitochondrial genes, mitochondrial DNA content, and the key transcription factor Glis of the Hh signaling pathway. (**F**) Western blotting analysis of the expression of the key transcription factor Glis of the Hh signaling pathway and browning thermogenic gene expression. (**G**) The resulting quantity protein level. Scale bar = 20 μm, * *p* < 0.05; ** *p* < 0.01.

In order to further verify the effect of leptin and the Hh signaling pathway on adipocyte browning, mice iWAT cells were used in this experiment, and browning induction was used to induce cell differentiation. At the same time, cells were treated with 100 nmol/L of leptin or 10 nmol/L of Pu, or 100 nmol/L of leptin and 10 nmol/L of Pu, in differentiation medium II. The morphology of the cells was noted with an inverted microscope, and the cells were harvested for oil red O after 8 d (Figure 6A). Compared with NC, the lipid droplets were bigger, as seen by the naked eye after the cells were treated with Pu, and leptin reversed the effects of Pu on the lipid droplets. In order to investigate the reasons for the decrease of the lipid droplets, RT-qPCR analysis was undertaken in this study, and showed that leptin significantly increased the expression of thermogenic genes and mitochondrial genes, and recovered Pu’s inhibition of these genes (*p* < 0.05 or *p* < 0.01) (Figure 6B,C), and leptin significantly increased mitochondrial DNA copy numbers (*p* < 0.05 or *p* < 0.01) (Figure 6D). However, leptin significantly decreased the expression of *Gli1/2/3*, the Hh signaling pathway key transcription factor genes (*p* < 0.05) (Figure 6E). The results of western blotting were consistent with the RT-qPCR results (Figure 6F,G). Leptin significantly increased the expression of thermogenic genes and mitochondrial genes, and the mitochondrial DNA copy numbers, but significantly decreased the Hh signaling pathway transcription factor genes. These results expounded that leptin promotes white adipose browning by inhibiting the Hh signaling pathway.

## 4. Discussion

Loss of leptin (ob/ob) or the Lep-R mutation (db/db) causes a series of metabolic abnormalities in mice, including obesity, diabetes, and hypercorticoidemia. It can be seen that leptin is essential for maintaining the body energy balance. The Hh signaling pathway plays a vital role in embryogenesis. The Gli transcription factor family plays an important role in the Hh signaling pathway. Gli1 and Gli2 mainly act as transcriptional activators, even though Gli2 has a weak repressing activity. Gli3 mainly functions as a transcriptional repressor [33,34,35]. Therefore, the expression level of Glis indirectly reflects the inhibition and activation of Hh pathway signaling. In addition to white and brown adipose tissue, there is another kind of adipose tissue—beige adipose tissue [36]—that is similar to brown adipose. However, the origin of beige adipose tissue remains controversial. Recent studies have shown that beige adipose cells are not only derived directly from the pre-adipocytes that do not express myf5, but are also converted from white adipose cells [37] in a process known as white adipose browning. Research shows that several factors could affect the browning of white adipose tissue, such as PPARγ [38], PRDM16 [39], PGC1α, and Irisin [40]. White adipose browning can increase energy consumption, reduce weight [41], increase insulin sensitivity [42], and improve glucose tolerance [43] and cardiovascular disease [44].

Chen et al found that leptin was not able to treat typical obesity; however, it is effective for reversing leptin deficiency-induced obesity, and was found to be possibly useful in treating lipodystrophy [45]. In order to study the role of leptin in white adipose browning, we injected leptin into HFD-fed mice. It was found that leptin acute injections decreased body weight and inhibited food intake in the mice. These results were consistent with Jeffrey et al discovery that leptin could regulate food intake [46]. Interestingly, *Gli1* expression was decreased in iWAT, indicating leptin was related to the Hh signaling pathway.

Zhang et al discovered that Hh signaling regulated PthrP and adiponetic secretion in bone, and PthrP and adiponetic regulated whole body energy metabolism by promoting white adipocyte browning [8]. Farr et al [47] showed that activation of Hh signaling, specifically in the brown adipose (BAT) of mice during development, resulted in the loss of interscapular BAT. These studies indicated that the Hh signaling pathway had an important role in energy metabolism and white adipocyte browning. Therefore, we used Pu or Cy to specifically activate or inhibit the Hh signaling pathway, and found that activation of the Hh signaling pathway inhibited the expression of thermogenic related genes and mitochondrial genes, but the expression levels of key genes of the Hh signaling pathway were increased. Our data further show that the Hh signaling pathway inhibited white adipocyte browning.

In addition, after treatment with different concentrations of leptin recombinant protein, the expression of *Gli1*, the key transcription factor of the Hh signaling pathway, was decreased with the increase of leptin. This indicates that leptin inhibited the Hh signal pathway. With inhibition of the Hh signaling pathway, the expression of thermogenesis and mitochondrial related genes was increased.

To illustrate the relationship between leptin and Hh signaling, the cells were treated with leptin. Compared with NC, the lipid droplets were smaller and the number of cells was larger. This showed that leptin reduces the lipid droplet size of adipocytes. Similarly, the same results can be obtained with Cy treatment, so it was speculated that the role of leptin may be consistent with Cy inhibition of the Hh signaling pathway. To confirm this speculation, we tested the expression of Gli1, a key factor of the Hh signaling pathway, and found that leptin inhibited the expression of Gli1. After Pu with leptin treatment compared with only Pu treatment, *Gli1* expression was reduced in the mRNA and protein levels. This suggested that the role of leptin was consistent with it inhibiting the Hh signaling pathway. However, Liu et al. found that leptin can activate the Hh signaling pathway in 3T3-L1 cells to inhibit adipocyte differentiation [8]. We speculate that leptin plays a different role in fat differentiation and browning; however, confirmation of this requires further research. Previous studies have shown that leptin acts on POMC neurons to promote BAT activation and white adipocyte browning [46]. In this experiment, compared with the NC group, leptin significantly increased the expression of *UCP1* and *PGC1α* in the mRNA and protein levels. This demonstrates that leptin significantly enhanced white adipocyte browning, which is consistent with Dodd’s results [48].

## 5. Conclusions

**Figure 7:**
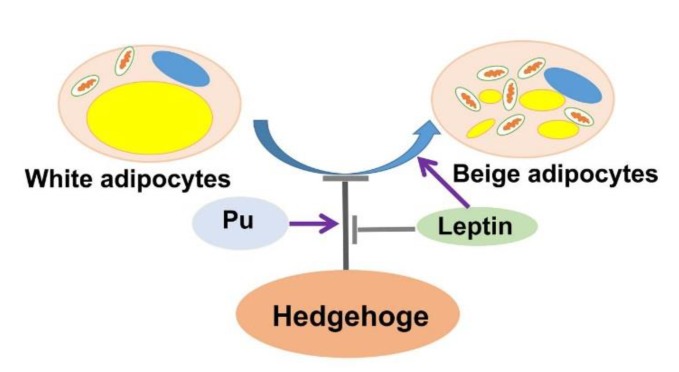
Graphical display of the interplay between leptin and the Hh signaling pathway in adipocyte browning.

In summary, in this study, leptin was considered to promote white adipose browning and inhibit the Hh signaling pathway. It was found that activation of the Hh signaling pathway inhibited white adipocyte browning, while its inhibition promoted white adipocyte browning. This study further showed that leptin reversed the effects of Pu on white adipose browning. The above results show that leptin promotes adipocyte browning by inhibiting the Hh signaling pathway (Figure 7). However, the elaborate mechanism through which leptin regulates white adipocyte browning via Hh signaling remains to be clarified through further study.

## Figures and Tables

**Table 1:** Sequences of primers for RT-qPCR in this study.

Gene	Forward(5′–3′)	Reverse(5′–3′)
*Gli1*	CAAGGCCTTTAGCAATGCCAGTGA	ATGCACTGTCTTCACGTGTTTGCG
*Gli2*	GGCAGCTTGCATCTTGAAG	AAAAAGCTCTGAAAACTCGTCCAT
*Gli3*	CCAGCCGAAAACGTACACTGT	GGGATGTTCTTATCATGGTCTGAA
*PPARγ*	AGGGCGATCTTGACAGGAAAGACA	AAATTCGGATGGCCACCTCTTTGC
*aP2*	ACACCGAGATTTCCTTCAAACTG	CCATCTAGGGTTATGATGCTCTTCA
*UCP1*	AGCCACCACAGAAAGCTTGTCAAC	ACAGCTTGGTACGCTTGGATACTG
*PGC1α*	GTCAACAGCAAAAGCCACAA	TCTGGGGTCAGAGGAAGAGA
*PRDM16*	TCATATGCGAGGTCTGCCACAAGT	TAGTGCTGAACATCTGCCCACAGT
*Cidea*	TGCTCTTCTGTATCGCCCAGT	GCCGTGTTAAGGAATCTGCTG
*Cox7a*	GTCTCCCAGGCTCTGGTCCG	CTGTACAGGACGTTGTCCATTC
*16S*	CCGCAAGGGAAAGATGAAAGAC	TCGTTTGGTTTCGGGGTTTC
*Hk2*	GCCAGCCTCTCCTGATTTTAGTGT	GGGAACACAAAAGACCTCTTCTGG
*β-tubulin*	GGGAGGTGATAAGCGATGAA	CCCAGGTTCTAGATCCACCA
